# Microbiological Production of Isocitric Acid from Biodiesel Waste and Its Effect on Spatial Memory

**DOI:** 10.3390/microorganisms8040462

**Published:** 2020-03-25

**Authors:** Igor G. Morgunov, Svetlana V. Kamzolova, Olga V. Karpukhina, Svetlana B. Bokieva, Julia N. Lunina, Anatoly N. Inozemtsev

**Affiliations:** 1 Federal Research Center, Pushchino Scientific Center for Biological Research of the Russian Academy of Sciences, G.K. Skryabin Institute of Biochemistry and Physiology of Microorganisms of the Russian Academy of Sciences, Prospect Nauki 5, Pushchino, 142290 Moscow Region, Russia; luninaju@rambler.ru; 2 Department of Higher Nervous Activity, Faculty of Biology, Lomonosov Moscow State University, 1-12 Leninskie Gory, 119234 Moscow, Russia; karpukhina.msu@yandex.ru (O.V.K.); a_inozemtsev@mail.ru (A.N.I.); 3 Department of Anatomy, Physiology and Botany, Khetagurov North Ossetian State University, 44-46 Vatutina str, 362025 Vladikavkaz, North Ossetia, Russia; bokievasb@rambler.ru

**Keywords:** (2*R*,3*S*)-isocitric acid, *Yarrowia lipolytica*, biodiesel waste, optimization, rats, heavy metals, stress, learning, memory

## Abstract

Within this work, the microbial synthesis of (2*R*,3*S*)-isocitric acid (ICA), a metabolite of the nonconventional yeast *Yarrowia lipolytica*, from biodiesel waste, has been studied. The selected strain *Y. lipolytica* VKM Y-2373 synthesized ICA with citric acid (CA) as a byproduct. This process can be regulated by changing cultivation conditions. The maximal production of ICA with the minimal formation of the byproduct was provided by the use of a concentration of (NH_4_)_2_SO_4_ (6 g/L); the addition of biodiesel waste to cultivation medium in 20–60 g/L portions; maintaining the pH of the cultivation medium at 6, and degree of aeration between 25% and 60% of saturation. Itaconic acid at a concentration of 15 mM favorably influenced the production of ICA by the selected strain. The optimization of cultivation conditions allowed us to increase the concentration of ICA in the culture liquid from 58.32 to 90.2 g/L, the product yield (Y) by 40%, and the ICA/CA ratio from 1.1:1 to 3:1. Research on laboratory animals indicated that ICA counteracted the negative effect of ammonium molybdate (10^−5^ M) and lead diacetate (10^−7^ M) on the learning and spatial memory of rats, including those exposed to emotional stress.

## 1. Introduction

The interest in isocitric acid (ICA) is associated with its potential efficiency in the treatment of Parkinson’s disease, Fe-deficiency anemia, and metabolic myopathies associated with a shortage of succinate dehydrogenase and aconitate hydratase, as well as the mutation of the gene of NADP-dependent isocitrate dehydrogenase 1 (*IDH1*) as one possible reason for the emergence of neoplasms [1,2,3,4,5,6]. In experiments with oxidative stress induced by the action of hydrogen peroxide and ecotoxic heavy metals (Cu, Pb, Zn, Cd) on the *Paramecium caudatum* infusoria cells, the monopotassium salt of ICA was found to be a more efficient antioxidant than ascorbic acid [7]. ICA monopotassium salt also mitigates the neurotoxic effect of lead and molybdenum salts, as is evident from its beneficial effect on the learning and memory inhibition in rats intoxicated with these heavy metal salts [8].

ICA is commonly produced by chemical synthesis with the formation of a mixture of stereoisomers extremely persistent for separation. The naturally occurring stereoisomer (2*R*,3*S*)-ICA produced by Sigma (USA) is isolated from the juice of the specially cultivated plant *Sedum spectabile*.

Recently, there is increasing interest in the production of ICA by microbiological methods, because the end product predominantly contains the natural ICA isomer and has a high biological activity [1,2,3,4,5,6].

The promising ICA-producing strains of the nonconventional yeast *Yarrowia lipolytica* were either isolated from natural sources [9,10,11,12,13,14,15], or were derived by classical mutagenesis [9,14,16], or were obtained by gene engineering technique by overexpression of the *ACO1* gene coding aconitate hydratase or the overexpression/inactivation of the *ICL1* gene coding isocitrate lyase [3,13,17,18]. The most developed processes of ICA production with the aid of *Y. lipolytica* strains include their cultivation on *n*-alkanes, ethanol, or various vegetation oils and are characterized by the accumulation of 80–110 g/L ICA after 5–6 days of fermentation with the yield (Y_ICA_) = 0.64–0.90 g/g substrate consumed [8,9,11,14,15,16].

To reduce the production cost of ICA, researchers try to use cheap and renewable carbon sources, such as crude glycerol, which is formed in large amounts as a by-product of biodiesel production. The annual production of biodiesel reaches 41 million tons and elevates annually by 4.5%. Each ton of produced biodiesel gives rise to 100 kg of biodiesel-derived waste glycerol, which poses the problem of its utilization [19,20]. The main disadvantage of the biodiesel-derived waste glycerol application as a carbon substrate is associated with variations in its composition depending on the biodiesel production process and feedstock used. Typically, biodiesel-derived waste glycerol contains 65–85% glycerol and impurities such as free fatty acids (1–5%, *w*/*w*), sodium, or potassium salts (4–12%, *w*/*w*), methanol (0.1–3%, *w*/*w*), heavy metals and other organic substances (0.5–0.8%, *w*/*w*) [21,22,23,24]. However, in some samples the amount of fatty acids can be quite high, in particular, it reaches 60% of impurities in biodiesel-derived waste obtained from waste vegetable oil [25]. Since the purification of biodiesel-derived waste glycerol is an expensive process, the valorization of biodiesel-derived wastes in the microbiological industry to produce valuable products is of great environmental and economic importance.

With the aid of *Y. lipolytica* strains, biodiesel waste can be converted into microbial protein [26], lipids [27,28,29,30], polyols [31,32], ketoacids [33,34], as well as citric acid (CA) and its salts [35,36,37,38]. In addition to CA, the wild strains *Y. lipolytica* cultivated on biodiesel waste accumulates ICA in sufficient amounts [39,40]. According to literature data, the best results of the fermentation of biodiesel waste into ICA (42.5 g/L ICA with its content reaching 56% of the ICA+CA sum) were obtained with a genetically engineered *Y. lipolytica* strain with the superexpressed *Gut1* and *Gut2* genes [41].

The aim of this work was to determine cultivation conditions promoting the production of ICA by the yeast *Y. lipolytica* grown on biodiesel waste as the carbon source, as well as to study the effect of ICA isolated from the culture liquid on the spatial memory of laboratory rats. 

## 2. Materials and Methods

### 2.1. Microorganisms

Experiments were carried out with three yeast strains of different types (Table 1). The wild strain *Y. lipolytica* VKM Y-2373 was selected as an ICA producer using cultivation medium with rapeseed oil [14]. The mutant strain *Y. lipolytica* UV/NNG was derived from the wild strain *Y. lipolytica* VKM Y-2373 by a combined treatment with UV light (5 min) and N-methyl-N’-nitro-N-nitrosoguanidine (50 µg/mL). The mutant was unable to grow in media with acetate as the carbon source; it synthesized isocitrate and citrate at a ratio of 2.7:1 on rapeseed (canola) oil [16]. The recombinant strain *Y. lipolytica* no. 20 with the superexpressed *ACO1* gene of aconitate hydratase was derived from the wild strain *Y. lipolytica* 607. The transformation of the wild strain increased the ICA:CA ratio from 1.1:1 to 2.3:1 [18].

### 2.2. Chemicals

All chemicals were of analytical grade (Sigma-Aldrich, St. Louis, MO, USA). The biodiesel waste used as the substrate contained (%): glycerol, 70.8; fatty acids, 23.9; sodium salts, 4; methanol, 0.3; and other organic compounds, 0.8. The content of metal ions consisted of (ppm): Cu, 0.3; Mg, 100; Fe, 13.7; Zn, 2.9; Ca, 46. The content of the heavy metals Cr, Cd, and Hg in the substrate was ≤0.01 mg/L. The amount of glycerol and fatty acids in waste were analyzed as described below.

### 2.3. Cultivation Conditions

In order to obtain yeast cells for the inoculation of fermenter, a 750 mL Erlenmeyer flask containing 100 mL of cultivation medium (see below) was inoculated with a yeast colony grown on the agar medium. The flask was incubated on an orbital shaker (130 rpm) at 29 ± 1 °C for 48 h (to a cell mass density of 5–6 g/L). At regular intervals, the pH of the medium was adjusted to a value of 4.5–6.0 by adding an appropriate volume of 10% KOH. 

The strains under study were cultivated in a 10-L ANKUM-2 M fermenter containing 5 L of fermentation medium. The cultivation temperature was 29.0 ± 0.1 °C, the concentration of dissolved oxygen (pO_2_) was equal to 55–60% saturation. The pH of the medium during fermentation was maintained at 6.0 by adding the necessary volume of 22% KOH solution. The medium contained (g/L): (NH_4_)_2_SO_4_, 3.0; MgSO_4_·7H_2_O, 1.4; Ca(NO_3_)_2_, 0.8; NaCl, 0.5; KH_2_PO_4_, 2.0; K_2_HPO_4_, 0.2; double volume of Burkholder microelement solution with slight modifications (in mg/L): KI—0.1, B^3+^ 0.01, Fe^2+^ 0.05, Zn^2+^ 0.04, Mn^2+^ 0.01, Cu^2+^ 0.01, Mo^2+^ 0.01; yeast autolysate, 8 mL/L; thiamine, 100 µg/L.

In experiments for ICA production of the studied strains and the effect of nitrogen the initial medium containing 20 g/L of biodiesel waste as the growth substrate. During cultivation, when pO_2_ of the medium exceeded its normal value by 5–10% (indicating the exhaustion of both glycerol and fatty acids in the composition of biodiesel waste), its concentration was replenished to 20 g/L. In experiments on the effect of the concentration of biodiesel waste, the medium initially contained from 20 to 150 g/L of substrate; with a decrease in pO2 of 5–10%, a similar amount of waste was added to the medium. To study the effect of pH, itaconic acid and aeration on acid formation and the fermentation medium of the producing strain (which initially contained 40 g/L biodiesel waste) was additionally supplemented with 60 g/L biodiesel waste after 2 days of cultivation.

Cultivation lasted 6 days.

The effects of pH, aeration, and the concentrations of nitrogen source, biodiesel waste, and itaconic acid on the production of ICA by *Y. lipolytica* VKM Y-2373 were studied in experiments with the fed-batch cultivation of this strain in the ANKUM-2M fermenter (SKB, Pushchino, Russia). These experiments are described in more detail in the Results and Discussion section.

### 2.4. Analyses

The concentrations of biomass, CA, ICA, and other organic acids (pyruvic, α-ketoglutaric, succinic, fumaric, and malic), as well as enzyme activities, are described in detail elsewhere [14].

Glycerol in the filtrate was analyzed by gas-liquid chromatography (GLC) on a Chrom-5 chromatograph (Czech Republic) with a flame-ionization detector using a glass column (200 × 0.3 cm) packed with 15% Reoplex-400 on Chromaton N-AW (0.16–0.20 mm) at a column temperature of 200 °C; argon was used as a carrier gas. Glycerol concentration was estimated by using a calibration curve.

Fatty acids in the filtrate was extracted twice with n-hexane. The filtrate-hexane mixture was allowed to stay and separate into two phases. The upper phase contained extracted lipids in n-hexane, while the lower aqueous phase did not contain them. The hexane extract was collected into a glass flask and dried by passing it through a glass filter with anhydrous sodium sulphate; the solvent was evaporated to constant mass.

### 2.5. Calculation of Fermentation Parameters

To take into account the medium dilution due to the addition of KOH solution for maintaining the constant pH value, the total amounts of ICA in the culture liquid were used for calculations.

The mass yield coefficient of ICA production (*Y*), expressed in g of ICA per g of glycerol and fatty acids consumed, was calculated from:

$$Y=\frac{P}{S}$$

The volume productivity (*Q*), expressed in g/L·h, was calculated from:

$$Q=\frac{P}{V\cdot t}$$

where *P* is the total amount of ICA in the culture liquid at the end of cultivation (g), *S* is the total amount of glycerol and fatty acids consumed (g), *V* is the initial volume of culture liquid (L), and *t* is the fermentation duration (h).

### 2.6. Isolation and Purification of ICA

ICA was isolated from the culture liquid as a monopotassium salt. After purification, isocitrate represented white crystalline powder with a purity of 99.9% [7].

### 2.7. Testing of ICA

Experiments on laboratory animals were carried out according to the rules of European Convention for the Protection of Vertebrate Animals used for Experimental and Other Scientific Purposes (Strasbourg, 1986) and of Directive 2010/63/EU of the European Parliament and of the Council of 22 September 2010 on the protection of animals used for scientific purposes.

The rats were purchased from the Central Nursery of Laboratory Animals “Stolbovaya” (Russia).

The experiments were carried out on 5 groups of animals, each group containing 10 outbred white male rats at the age of 4 months at the beginning of the experiment. The rats were kept in plastic cages at a constant temperature (+21, +22 °C) at a 12-hour light day and had unlimited access to water and food.

The animals of the first group were given a dose of 20 mg ICA/kg; the animals of the second group were given a dose of 10^−5^ M ammonium molybdate; the animals of the third group were given ICA in combination with 10^−7^ M lead diacetate; the animals of the fourth group were given ICA in combination with ammonium molybdate; and the animals of the fifth group were the control. Aqueous solutions of heavy metal salts were injected intraperitoneally 5 h before the beginning of the experiment (moment of stimuli application) in a volume of 2 mL. ICA solution was injected in the same volume 4 h after the injection of heavy metal salt solutions. The control rats were injected with distilled water (2 mL) one hour before the experiment.

### 2.8. Statistical Analysis

All the data of biosynthetic experiments are the mean values of three experiments and two measurements for each experiment; the standard deviations were calculated (SD < 10 %).

In animal experiments, the data were analyzed with one-way ANOVA followed by post-hoc Tukey’s test. The statistical significance was set at *p* < 0.05. The data are shown as mean and standard error of the mean.

## 3. Results and Discussion

### 3.1. Production of ICA

Parameters characterizing the growth of strains under study and synthesis of ICA and other acids are shown in Table 1. All three strains grew relatively well on the biodiesel waste and synthesized citric acids with the ICA/CA ratio between 1.1:1 and 1.3:1. The maximum amount of biomass (12.67 g/L) was accumulated by the wild strain *Y. lipolytica* VKM Y-2373, while the biomasses accumulated by the mutant strain *Y. lipolytica* UV/NNG and the recombinant strain *Y. lipolytica* no. 20 (*ACO1*) were lower by 36% and 29%, respectively. The poorer growth of the modified strains may be related to their higher sensitivity to impurities present in the waste glycerol. As a rule, the contaminating methanol does not influence the cellular membrane of growing microorganisms. In contrast, the free fatty acids (linoleic, stearic and oleic) and monovalent mineral salts present in sufficient amounts in the biodiesel-derived waste glycerol negatively affect the diffusion of necessary nutrients through the cellular membrane and thus reduce their availability [42,43,44].

As seen from Table 1, the wild strain VKM Y-2373 accumulated a higher amount of ICA (58.32 g/L) then the mutant UV/NNG and the transformant no. 20 (*ACO1*) (44.6 and 33.6 g/L, respectively). On the other hand, acid production by the transformant and especially mutant shifted towards ICA (ICA/CA = 1.2:1 and 1.3:1, respectively, in comparison with 1.1:1 typical of the wild strain). In addition to CA and ICA, the strains excreted other organic acids (pyruvic, α-ketoglutaric, succinic, fumaric, and malic), whose content mas the maximum for the mutant. All further experiments were carried out with the wild strain because it showed the maximum values of the ICA yield (*Y* = 0.79 g/g) and volume productivity (*Q* = 0.62 g/L∙h).

Table 2 shows the effect of (NH_4_)_2_SO_4_ as the nitrogen source on the growth of *Y. lipolytica* VKM Y-2373 and the synthesis of ICA by this strain. At a low concentration of 1.5 g/L, (NH_4_)_2_SO_4_ provided neither good growth nor good ICA production. At a high concentration of 10 g/L, ammonium sulfate provided good growth and relatively good ICA production. However, the best results were obtained when ammonium sulfate was added to the cultivation medium in the concentration 6 g/L. In this case, the strain accumulated the maximum amount of ICA (70.22 g/L) with the maximum ratio ICA/CA = 1.5:1.

Earlier, the positive effect of (NH_4_)_2_SO_4_ on ICA production was also observed for another strain *Y. lipolytica* IMUFRJ 50682, which accumulated 16.79 g/L ICA and 1.46 g/L CA in the cultivation medium supplemented with ammonium sulfate in comparison with 6.92 g/L ICA and 8.21 g/L CA accumulated in the medium without ammonium sulfate [39]. The mechanism of the positive effect of ammonium sulfate on ICA productions needs further studies at the level of enzymes involved in the metabolism of ICA. It should be noted in this regard that the activity of isocitrate dehydrogenase (EC 1.1.1.42), an enzyme that catalyzes the conversion of isocitrate into α-ketoglutarate in the tricarboxylic acid (TCA) cycle, decreases by 66% in response to the excess of NH4^+^ ions in the cultivation medium of the nitrogen-fixing bacterium *Azospirillum lipoferum* [45].

Further experiments were carried out with the optimal concentration of (NH4)_2_SO_4_ in the cultivation medium equal to 6 g/L.

The effect of biodiesel waste on ICA production was studied using the substrate in concentrations between 20 and 150 g/L (Table 3).

As seen from this Table 3, in all experiments, *Y. lipolytica* VKM Y-2373 realizes the concurrent uptake of glycerol and the fatty acid fractions during the conversion of biodiesel waste, although glycerol was utilized at a higher rate than fatty acids. These data are comparable with those [28] obtained for *Y. lipolytica* cultivated on mixtures of saturated free fatty acids (an industrial derivative of animal fat called stearin) and technical glycerol (the main by-product of bio-diesel production facilities). As the concentration of biodiesel waste varied from 20 to 80 g/L, it does not inhibit the glycerol and fatty acids uptake. With the further increase in the concentration of added waste (up to 150 g/L), the glycerol and fatty acids uptake decreased twofold. When the medium was supplemented with 20 g/L biodiesel waste, the strain accumulated 70.22 g/L ICA with the ICA/CA ratio equal to 1.5:1. In the medium containing 40 to 80 g/L biodiesel waste, the strain produced ICA in concentrations between 76.65 and 80.12 g/L, with the maximum value of the ICA/CA ratio equal to 2:1. Still, higher biodiesel waste concentrations (100 and 150 g/L) suppress the growth of the strain and its production of CA and especially ICA, so that the ICA/CA ratio lowered to 1.1:1. In our opinion, high concentrations of biodiesel waste in the medium, such as 100 and 150 g/L, may suppress the growth of *Y. lipolytica* VKM Y-2373 and its production of acids because of high foam formation. As a result, the yeast cells concentrated in the foam layer are excluded from the process of acid formation. 

As for literature data on the effect of high concentrations of biodiesel waste, they are contradictory. Thus, the increase in the content of biodiesel waste in the cultivation medium of *Y. lipolytica* IMUFRJ 50682 from 45 to 160 g/L shifted the ICA/CA ratio from 0.5:1 to 1.2:1 [39]. At the same time, other researchers recommend avoiding high concentrations of waste glycerol in the medium and add it in portions of 20–50 g/L as it is consumed [33,34].

As seen from Table 4, the strain grew well at pH values from 3 to 6. At higher pH values (6.5 and 7.0), the accumulated biomass was slightly lower, presumably because of culture dilution with the KOH solution used for maintenance of the culture pH. The production of acids took place within a wide range of pH values (from 3 to 7), the maximum production of ICA being observed at pH 6.0 and 6.5 (80.12 and 76.3 g/L ICA, respectively, with the ICA/CA ratio equal to 2:1 and 2.5:1). These results are in agreement with those obtained for the wild yeast *Y. lipolytica* cultivated on ethanol or rapeseed oil [14,15], but contradict the results of the study of ICA production by the genetically modified *Y. lipolytica* strain with the superexpressed *Gut1* and *Gut2* genes, which efficiently produced both CA and ICA at acidic pH values and hence did not require the maintenance of the culture pH during cultivation [41]. 

In further studies, the pH of the culture medium was maintained at a level of 6.0.

Information on the pathway of ICA formation by wild and genetically modified microbial strains cultivated on n-alkanes, ethanol, or vegetable oils can be found in the literature [9,11,13,14,15,46]. The authors of these papers emphasize the key role of isocitrate lyase, an enzyme of the glyoxylate cycle involved in the metabolism of ICA. The inhibition of isocitrate lyase with itaconic or oxalic acids, structural analogues of succinate and glyoxylate, shifted the process of acid formation towards the preferential formation of ICA. The best results were observed with itaconic acid added to the medium at a concentration of 30–40 mM [46]. 

The effect of itaconic acid on the production of ICA by yeasts (specifically, *Y. lipolytica* VKM Y-2373) in the case of cultivation on biodiesel waste was first studied in this work. As evident from the comparison of biotechnological parameters (ICA concentration, Y, Q and the ICA/CA ratio) shown in Table 5, itaconic acid at a concentration of 15 mM favorably influences the production of ICA by *Y. lipolytica* VKM Y-2373 from biodiesel waste. In this case, however, itaconic acid elevated the ICA/CA ratio only by 1.5 times (from 2:1 to 3:1), whereas, in the case of ICA production from rapeseed oil, itaconic acid increased this ratio by 6 times [14].

To understand the reason of less efficiency of itaconic acid in the production of ICA from biodiesel waste, the activities of enzymes involved in the metabolism of ICA were measured in the *Y. lipolytica* VKM Y-2373 cells taken from the culture in the period of active acid formation (72 h of cultivation). The results of the enzyme assay are shown in the lower part of Table 5. As seen from this information, citrate synthase has a high activity (2.1–2.91 U/mg protein) at all concentrations of itaconic acid in the cultivation medium, indicating an important role of this enzyme in the synthesis of both CA and ICA acids. The activity of aconitate hydratase was also relatively high (0.63–0.83 U/mg protein), especially in comparison with that of NAD-dependent isocitrate dehydrogenase (NAD-ICDH) (0.02–0.06 U/mg protein). Makri et al. [47] also observed a low activity of NAD-ICDH during the biosynthesis of citric and isocitric acids in *Y. lipolytica* cultivated on glycerol under nitrogen limitation. The activity of isocitrate lyase was low (0.081 U/mg protein) even in the absence of itaconic acid in the cultivation medium, being almost zero (0.002 U/mg protein) in the presence of 60-100 mM itaconic acid. Thus, despite the almost complete inhibition of the activity of isocitrate lyase by itaconic acid, the proportion between ICA and CA did not shift towards ICA, as was observed in the case of yeast cultivation on ethanol [15] and rapeseed oil [14]. These data are in agreement with the results of the study of the genetically modified strain *Y. lipolytica* H222-41(JMP5)Z123 with the inactivated *ICL1* gene, which showed only a 4% increase in the ICA/CA ratio in comparison with the wild strain *Y. lipolytica* H222 [13]. 

Further experiments on the effect of aeration on the ICA production were carried out in the presence of 15 mM itaconic acid in the cultivation medium.

Literature data on the aeration effect are contradictory. The maximum yield of ICA in the *Y. lipolytica* culture grown on *n*-alkanes was observed only at high aeration (85–90% saturation) [9]. In the medium with ethanol, *Y. lipolytica* actively synthesized equal amounts of ICA and CA at high aeration (90–95% saturation); at an average level of aeration (60–65% saturation), the yeast predominantly produced ICA, while the synthesis of CA was suppressed; poor aeration (28%-30% saturation) suppressed both yeast growth and acid formation [12]. In the medium with waste glycerol, the optimal yield of ICA was observed at 60–70% saturation [32], while with pure glycerol no significant effect of pO_2_ was found on citrate production [48]. There are data that the agitation rates of 800 and 900 rpm and aeration rates within the range of 0.24–0.48 vvm generated the dissolved oxygen concentrations higher than 40%, which are the best for citric acid biosynthesis from glycerol [49]. According to our data [50], the requirement of the yeast *Y. lipolytica* in oxygen considerably depends on the concentration of iron ions in the cultivation medium. 

In this regard, the effect of aeration was studied by either adding or not adding Fe^2+^ ions to the cultivation medium. The concentration of Fe^2+^ ions was 1.2 mg/L because it is this concentration that provided the maximum yield of ICA during the cultivation of *Y. lipolytica* VKM Y-2373 on rapeseed oil or ethanol [14,15]. 

As seen from Table 6, the maximum production of ICA (90.2 g/L ICA; Y = 1.11 g/g substrate consumed; Q = 0.90 g/L·h) in the absence of iron ions in the cultivation medium is at pO_2_ = 55–60% saturation. At lower aeration (25–30% saturation), the production of ICA is lower by 20%. The decrease in pO_2_ to 5–10% diminishes both cell biomass and ICA production by 3.4 and 6.6 times, respectively. This dependence of biomass on pO_2_ suggests that the yeast growth at pO_2_ = 5–10% saturation is limited by oxygen. The presence of Fe^2+^ ions in the cultivation medium at pO_2_ =5–10% saturation stimulates both cell growth and ICA production by 3.3 and 2.8 times, respectively. This result suggests that the poor aeration and the absence of Fe^2+^ ions in the medium cause double limitation (by iron and oxygen) or, in other words, limitation by metabolically available energy. At pO_2_ between 25% and 60% saturation, the effect of iron ions on biomass and ICA production was negligible.

For further experiments on laboratory animals, ICA was isolated from the culture liquid in the form of monopotassium salt; the isolated and purified product consisted of 99.9% ICA.

### 3.2. Effect of ICA on the Spatial Memory of Rats

To study the effect of pharmacological agents on the learning and spatial memory of rats, the experimental model of the formation of the conditioned active avoidance response (CAAR) is commonly used [51,52]. However, this model is not always efficient, and researchers use various impacts on the central nervous system (CNS), such as maximal electroshock and hypoxia [53]. Researchers also use various functional CNS disorders not related to invasive physical impacts, among which are the experimental extremal disturbances of the definite causal and spatial relationship, which additionally allows for emotional stress effects to be studied [54].

Table 7 shows the temporal event sequence of the experiment. First, the conditioned active avoidance response (CAAR) was developed in the test rats by the daily application of stimuli (25 trials per day) in a shuttle box as described in the previous work [8]. The experimental chamber was equipped with an electrifiable floor and divided into two equal compartments by a wall with two holes. In this case, the far (relative the observer) hole in the wall between two box compartments was opened. Then, the developed CAAR was smashed as described in the previous work [8]. Afterwards, two experiments were carried out. Within the second experiment, after 25 trials, the developed CAAR was subjected to spatial alteration by closing the far hole and opening the near hole in the wall. Then, the CAAR was tested in 20 trials under these altered spatial conditions.

Analysis of the data listed in Table 8 shows that the reproduction of the CAAR in the control rats after spatial transformation dramatically decreased. By the end of the experiment, the CAAR values were close to the initial ones. ICA counteracted this inhibition, and the drop in the CAAR reproduction was not so dramatic as in the control rats. The reproduction of the CAAR returned to the initial level after 6-10 trials. Throughout the experiment, the CAAR values of tested rats were statistically higher than in the control. 

Similar to the earlier results [8], lead ions strongly inhibited the CAAR, which did not allow us to smash the CAAR and perform its subsequent spatial transformation. The injection of ICA prevented the lead-induced inhibition of the CAAR. As a result, the reproduction of the CAAR after spatial transformation was close to the control values (Table 8).

Molybdenum ions turned out to be less inhibitory than lead ions, meaning that test rats performed almost seven avoidance responses from 10 possible ones before spatial transformation. Nevertheless, the number of CAARs within the last five trials of response restoration was less than in the control (Table 8). ICA prevented the inhibitory effect of molybdenum, meaning that the number of avoidance responses were close to the control level before spatial transformation and exceeded it after the transformation.

The effects of molybdenum and ICA deserve special attention. As mentioned above, molybdenum inhibits the CAAR, their number being less than in control rats and rats treated with ICA. With the combined injection of molybdenum and ICA, the number of CAARs was higher than in the control rats and in rats treated with ICA alone. This result seems quite paradoxical because heavy metals usually reduce the activity of neurotropic substances. It should, however, be noted that a similar paradoxical effect was earlier observed upon the combined application of molybdenum and Semax (Met-Glu-His-Phe-Pro-Gly-Pro), an analog of the adrenocorticotropin fragment (4–10) [55]. Namely, molybdenum and Semax inhibited the development of the CAAR when applied separately and stimulated it when applied together.

As shown earlier [7,8], ICA displays antioxidant properties and prevents oxidative stress caused by heavy metals. In particular, ICA counteracts the negative effect of oxidative stress on the learning and memory of animals. According to recent research, oxidative stress can be caused not only by detrimental factors, such as heavy metals or hypoxia, but also under normal conditions, for example, upon cognitive loads on CNS. The formation of the CAAR induces oxidative stress too. The antioxidant agent carnosine diminishes the stress and accelerates learning [56,57].

The spatial transformation of the CAAR used in this study is a more difficult task for animals than the development of avoidance responses. The drug Semax having antioxidant activity enhances the reproduction of avoidance responses after spatial transformation [58]. Taking into account the above information, the results of this study suggest that the spatial transformation of the CAAR causes oxidative stress in the tested animals and that the observed beneficial effect of ICA is explained by its antioxidant activity.

## 4. Conclusions

Thus, the production of ICA by the wild strain *Y. lipolytica* VKM Y-2373 is maximum when the cultivation medium contains 6 g/L of (NH_4_)_2_SO_4_ as the nitrogen source; from 40 to 80 g/L of biodiesel waste as the source of carbon and energy; 15 mM of itaconic acid as the inhibitor of isocitrate lyase; and when the pH of the medium during cultivation is maintained at a level of 6.0–6.5. The requirement of this strain in oxygen depends on the concentration of iron ions in the medium. The strain *Y. lipolytica* VKM Y-2373 cultivated under such conditions in a fermenter with a waste glycerol synthesized 90.2 g/L ICA with a yield of 1.11 g/g and the ICA/CA ratio of 3:1. These results have considerable promise for the industrial production of ICA. It should be emphasized that the yeast *Y. lipolytica* and its metabolites are generally recognized as safe (GRAS) [59,60].

The obtained data on the positive effect of ICA on laboratory animals in the model of the learning and spatial memory are crucial for future studies. Oxidative stress underlies many neurodegenerative diseases, such as Alzheimer’s. In this case, patients suffer from spatial memory loss, compromising their quality of life. The search for effective antioxidants that can counteract oxidative stress will greatly improve such persons’ quality of life.

## Figures and Tables

**Table 1:** Parameters of growth and (2*R*,3*S*)-isocitric acid (ICA) production by *Y. lipolytica* strains.

Parameter	*Y. lipolytica*		
	VKM Y-2373	UV/NNG	*ACO1* no. 20
Biomass (g/L)	12.67 ± 1.15	8.10 ± 1.75	9.00 ±1.15
ICA (g/L)	58.32 ± 4.45	44.60 ± 2.15	33.60 ± 4.22
CA (g/L)	53.02 ± 4.15	34.31 ± 3.75	28.00 ± 2.10
ICA/CA ratio	1.1:1	1.3:1	1.2:1
Other acids (% of the sum)	3.35	10.00	4.65
Total amount of ICA (*P*) (g)	446.15	309.52	302.40
Glycerol + Fatty acids consumed (*S*) (g)	563.33	586.24	461.89
Y (g/g)	0.79	0.53	0.65
Q (g/L·h)	0.62	0.43	0.42

**Table 2:** Effect of nitrogen concentration on the growth of *Y. lipolytica* VKM Y-2373 and ICA production.

Parameter	Concentration of (NH_4_)_2_SO_4_ (g/L)			
	1.5	3.0	6.0	10.0
Biomass (g/L)	5.87 ± 1.00	12.67 ± 1.15	18.75 ± 2.15	32.67 ± 1.15
ICA (g/L)	13.3 ± 1.45	58.32 ± 3.45	70.22 ± 2.45	28.15 ± 3.45
CA (g/L)	13.1 ± 1.15	53.02 ± 2.15	46.81 ± 2.85	23.02 ± 2.15
ICA/CA ratio	1:1	1.1:1	1.5:1	1.2:1
Total amount of ICA (*P*) (g)	97.36	446.15	539.99	251.94
Glycerol + Fatty acids consumed (*S*) (g)	177.00	563.33	635.28	434.38
Y (g/g)	0.55	0.79	0.85	0.58
Q (g/L·h)	0.14	0.62	0.75	0.35

**Table 3:** Effect of the initial concentration of biodiesel waste on growth and ICA production by *Y. lipolytica* VKM Y-2373.

Parameter	Concentration of Biodiesel Waste (g/L)					
	20	40	60	80	100	150
Biomass (g/L)	18.75 ±2.15	18.30 ±3.10	17.40 ± 2.10	16.80 ± 1.10	12.90 ± 2.10	10.90 ± 3.14
Glycerol uptake rate (g/L·h)	0.74 ± 0.40	0.74 ± 0.40	0.74± 0.40	0.70 ± 0.26	0.560± 0,20	0.35 ± 0,15
Fatty acids uptake (g/L·h)	0.22 ± 0.04	0.25 ± 0.04	0.22 ± 0.05	0.23 ± 0.03	0.12 ± 0.03	0.11 ± 0.03
ICA (g/L)	70.22 ± 2.45	80.12 ± 2.00	78.20 ± 2.20	76.65 ± 3.40	32.65 ± 6.12	17.65 ± 2.12
CA (g/L)	46.81 ± 2.85	41.00 ± 3.12	39.00 ± 2.12	37.65 ± 3.12	31.00 ± 5.40	16.65 ± 2.40
ICA/CA ratio	1.5:1	2:1	2:1	2:1	1.1:1	1.1:1
Total amount of ICA (*P*) (g)	539.99	597.60	583.20	576.00	230.40	122.40
Glycerol + Fatty acids consumed (*S*) (g)	635.28	602.05	593.86	606.08	419.59	222.90
Y (g/g)	0.85	0.99	0.98	0.95	0.55	0.55
Q (g/L·h)	0.75	0.83	0.81	0.80	0.32	0.17

**Table 4:** Effect of pH on growth and ICA production by *Y. lipolytica* VKM Y-2373.

Parameter	pH					
	3.0	4.0	5.0	6.0	6.5	7.0
Biomass (g/L)	20.0 ± 2.65	19.0 ± 3.53	21.0 ± 2.25	18.30 ± 3.10	16.30 ± 2.80	14.00 ± 2.65
ICA (g/L)	20.8 ± 3.85	42.4 ± 3.15	40.4 ± 3.85	80.12 ± 2.00	76.3 ± 3.40	40.73 ± 2.00
CA (g/L)	34.7 ± 3.45	60.8 ± 3.89	67.7 ± 3.45	41.00 ± 3.12	30.5 ± 2.15	26.00 ± 3.12
ICA/CA ratio	0.6:1	0.7:1	0.6:1	2:1	2.5:1	2.2:1
Total amount of ICA (*P*) (g)	180.00	338.40	316.80	597.60	597.60	360.00
Glycerol + Fatty acids consumed (*S*) (g)	692.00	676.00	597.00	602.05	649.56	654.00
Y (g/g)	0.26	0.50	0.49	0.94	0.90	0.54
Q (g/L·h)	0.25	0.47	0.44	0.83	0.83	0.50

**Table 5:** Effect of itaconic acid on growth and ICA production by Y. *lipolytica* VKM Y-2373.

Parameter	Itaconic Acid (mM)				
	0	15	30	60	100
Biomass (g/L)	18.30 ± 3.10	19.25 ± 2.10	17.70 ± 1.75	16.45 ± 1.10	11.45 ± 3.20
ICA (g/L)	80.12 ± 2.00	90.2 ± 1.10	84.1 ± 2.00	68.75 ± 2.00	40.0 ± 3.65
CA (g/L)	41.00 ± 3.12	29.6 ± 1.10	27.8 ± 1.70	20.8 ± 2.70	12.5 ± 0.65
ICA/CA ratio	2:1	3:1	3:1	3.3:1	3.2:1
Total amount of ICA (*P*) (g)	597.60	648.00	576.00	511.20	273.60
Glycerol + Fatty acids consumed (S) (g)	602.05	584.43	586.53	620.65	588.86
Y (g/g)	0.94	1.11	0.98	0.82	0.46
Q (g/L·h)	0.83	0.90	0.80	0.71	0.38
Enzyme activity (U/mg protein)					
Citrate synthase	2.910	2.800	2.750	2.350	2.10
Aconitate hydratase	0.805	0.830	0.730	0.730	0.630
NAD-ICDH	0.060	0.064	0.031	0.022	0.020
Isocitrate lyase	0.081	0.010	0.008	0.002	0.002

**Table 6:** Effect of aeration on growth and ICA production by *Y. lipolytica* VKM Y-2373.

Parameter	Without Fe^2+^			With Fe^2+^ = 1.2 mg/L		
	pO_2_ (% Saturation)			pO_2_ (% Saturation)		
	5–10	25–30	55–60	5–10	25–30	55–60
Biomass (g/L)	3.50 ± 1.10	15.00 ± 1.10	19.25 ± 2.10	11.50 ± 1.10	16.25 ± 1.10	17.55 ± 1.55
ICA (g/L)	8.42 ± 2.50	72.22 ± 1.50	90.2 ± 1.10	24.00 ± 3.50	80.4 ± 4.10	85.72 ± 4.35
CA (g/L)	6.25 ± 14.00	24.44 ± 2.00	29.6 ± 1.10	8.10 ± 1.00	26.8 ± 1.10	28.6 ± 2.10
ICA/CA ratio	1.3:1	3:1	3:1	3:1	3:1	3:1
Total amount of ICA (P) (g)	72.00	475.20	648.00	168.00	604.8	604.8
Glycerol + Fatty acids consumed (S) (g)	681.84	529.43	584.43	636.38	615.86	572.75
Y (g/g)	0.11	0.89	1.11	0.26	0.98	1.06
Q (g/L·h)	0.10	0.66	0.90	0.23	0.84	0.84

**Table 7:** Experimental sequence.

Days	1–4	5	6	7
Process	Formation of the CAAR	CAAR smash	CAAR restoration	Spatial transformation of the CAAR

**Table 8:** Effect of ICA and heavy metal salts on the conditioned active avoidance response (CAAR) (% of the number of trials) before and after its spatial transformation.

Variant	Before	After			
	21–25	1–5	6–10	11–15	16–20
Control	100 *	60 ± 29.6	70 ± 17.3	70 ± 17.3	94 ± 6.3
ICA	94 ± 13.5 *	78 ± 14.8 ^a^	92 ± 10.3 ^a^	94 ± 9.7 ^a^	100
ICA + Pb	86 ± 16.5	80 ± 24.9	82 ± 14.8	94 ± 9.7	80 ± 18.7
Mo	68 ± 10.4 ^a^	75 ± 27.8	65 ± 20.7	73 ± 21.2	88 ± 14.9
ICA+Mo	95 ± 9.3 ^b^	90 ± 14.1 ^c^	98 ± 6.3 ^a,b^	88 ± 21.5 ^a,b^	100 ^b^

Data are the mean and standard deviations of 5 trials. * *p* < 0.05 relative the values after spatial transformation; ^a^ *p* < 0.05 relative the control; ^b^ relative the values with Mo; ^c^ *p* < 0.05 relative the values with ICA.
